# Re-evaluate the downgrade of subglottic secretion drainage in VAP prevention guidelines: a call for evidence-based transparency

**DOI:** 10.1017/ice.2025.10346

**Published:** 2026-04

**Authors:** Hamid Khosrowshahi

**Affiliations:** Department of Research Development, Flosure Technologies, LLC, Tarrytown, NY, USA

## Letter to the Editor

This letter responds to the 2022 practice recommendations from the Society for Healthcare Epidemiology of America (SHEA), the Infectious Diseases Society of America (IDSA), and the Association for Professionals in Infection Control and Epidemiology (APIC), and their decision to downgrade subglottic secretion drainage (SSD) endotracheal tubes (ETTs) from “Essential” to “Moderate.”^1^ This letter builds upon previous discourse, such as the letter by Lichtenthal,^2^ by introducing new evidence and highlighting further inconsistencies. I respectfully urge the authors to reconsider this decision, as it appears inconsistent with the existing evidence base and recent developments, having neglected to consider other excellent evidence-based and reliable published studies in their evaluation.

Since the last guidance in 2014, numerous meta-analyses have consistently affirmed SSD’s efficacy in reducing ventilator-associated pneumonia (VAP) incidence. Studies by Caroff et al.^3^ and Carrascosa et al.^4^ all conclude that SSD significantly reduces VAP. Although these meta-analyses consistently report that SSD’s impact on mortality is not statistically significant, this outcome is a known challenge for VAP prevention studies. As noted by Caroff et al.,^3^ achieving statistical significance for mortality in VAP prevention trials would require sample sizes of many thousands of patients. The consistent reduction in VAP incidence itself, however, remains clear. Furthermore, as the late critical care luminary Luciano Gattinoni argued, relying on mortality as the sole primary end point is an insufficient measure of an intervention’s effectiveness, especially for interventions that improve patient-centered outcomes without a direct impact on survival. This physiological-first perspective, published in JAMA^5^ underscores why focusing exclusively on mortality can be misleading and can overlook important patient benefits like reduced organ dysfunction and shorter ICU stays. It is strikingly inconsistent that for the 2022 guidance, the authors primarily referenced the corrected Carrascosa et al.^4^ paper to justify the downgrade, despite its conclusions on VAP incidence being consistent with Caroff et al.,^3^ a paper with shared authorship. This selective focus, given the broad body of evidence, warrants clarification.

Furthermore, a profound inconsistency exists within the broader institutional commitment to SSD. The Agency for Healthcare Research and Quality (AHRQ), in its “Making Healthcare Safer” initiatives, recognized SSD as an essential intervention for VAP prevention.^6^ In 2013, AHRQ awarded Johns Hopkins University a substantial $7.3 million contract to identify best evidence-based VAP prevention interventions.^7^ Johns Hopkins’ subsequent publications, including key studies co-authored by members of the 2022 guidance committee (eg, Speck et al.^8^; Rawat et al.^9^), consistently identified and recommended SSD as a main and essential component of VAP prevention bundles. It is deeply concerning that some of the very clinicians involved in these AHRQ-funded Johns Hopkins studies, which supported SSD’s essential role, subsequently participated in guidance that downgraded SSD. This contradiction, where institutional findings appear to diverge from new guidance, demands a clear explanation.

This decision, if uncorrected, risks undermining efforts to achieve true “zero harm” by discouraging the adoption of a highly effective, cost-effective intervention, thereby perpetuating preventable patient suffering and increasing healthcare expenditures.

Finally, we must emphasize that VAP is a misnomer; the ETT is the “real culprit” for this infection, not primarily the mechanical ventilation itself. As a recent review by Lichtenthal and Borg^10^ states, the ETT disrupts natural protective barriers and facilitates the microaspiration of colonized secretions, which is the primary mechanism leading to VAP. This fundamental understanding of VAP’s pathogenesis underscores the critical importance of interventions like SSD that directly address the ETT’s role in preventing infection at its source.

Given the consistent evidence for SSD’s efficacy in VAP incidence reduction, the historical inconsistencies within a major institution’s publications, I respectfully urge the guidance committee to re-evaluate its 2022 guidance and restore SSD to its “Essential” classification. This revision is vital for ensuring that clinical guidelines align with the strongest available evidence, promoting transparency, and ultimately advancing patient safety.
